# The conquest of the north continues: *Baylisascaris procyonis* in free-ranging invasive raccoons (*Procyon lotor*) from Germany, including a first report in the northeastern state of Mecklenburg-Western Pomerania

**DOI:** 10.1016/j.ijppaw.2025.101139

**Published:** 2025-09-17

**Authors:** Zaida Rentería-Solís, Luis Flores, Torsten Langner, Sandra Gawlowska, Thomas Grochow, Simone Fietz, Stefan Birka, Nina Król, Anna Obiegala

**Affiliations:** a Institute of Parasitology, Centre for Infection Medicine, Faculty of Veterinary Medicine, Leipzig University, An den Tierkliniken 35, 04103 Leipzig, Germany; b Friedrich-Loeffler-Institute, Federal Research Institute for Animal Health, Südufer 10, 17493, Greifswald-Insel Riems, Germany; c Clinic for Ruminants and Swine, Faculty of Veterinary Medicine, Leipzig University, An den Tierkliniken 11, 04103, Leipzig, Germany; d Kuhkraft – gute gesunde Kühe, Veterinary Services, Saalenstr. 2, 35110, Frankenau-Ellershausen, Germany; e Institute of Food Hygiene, Faculty of Veterinary Medicine, Leipzig University, An den Tierkliniken 1, 04103, Leipzig, Germany; f Institute of Veterinary Anatomy, Histology and Embryology, Faculty of Veterinary Medicine, Leipzig University, An den Tierkliniken 41-43, 04103, Leipzig, Germany; g Institute of Animal Hygiene and Veterinary Public Health, Faculty of Veterinary Medicine, Leipzig University, An den Tierkliniken 1, 04103, Leipzig, Germany; h Institut d’Ecologie et des Sciences de l’Environnement Paris (IEES), Sorbonne Université, 4, place Jussieu, 75002 Paris cedex 5, France

**Keywords:** Raccoon roundoworm, Zoonoses, Nematode, Invasive species, *Baylisascaris procyonis*

## Abstract

With exception of the Northeast, the raccoon roundworm (*Baylisascaris procyonis*) is widespread in Germany. This zoonotic parasite can cause neurological disease in paratenic and aberrant hosts, like humans. As the name indicates, raccoons are the definitive host of *B. procyonis*. However, and despite the successful expansion of the raccoon population, parasite and host ranges do not always overlap. *B. procyonis* has been largely absent from the northeastern part of the country, notwithstanding the stable presence of raccoons in this area. In this study, faecal and intestinal samples were opportunistically collected from 166 free-ranging raccoons from 9 federal states in Germany. In 68 animals (41.0 %), *B. procyonis* was identified either through PCR or morphological identification of adult worms. The positive raccoons originated from 6 federal states, including for the first time animals from the northern state of Mecklenburg-Western Pomerania. The results of this study highlight the dissemination of the parasite in the north of the country, while maintaining its presence in the rest of Germany.

## Highlights

• *Baylisascaris procyonis* is widespread in Germany.
• We examined samples of 166 raccoons from Germany.
• *B. procyonis* was found in 41 % of the raccoons, including animals from northern state of Mecklenburg-Western Pomerania.
• Our results indicate that the parasite is expanding into new areas in Germany.

## Graphical abstract

**Figure:**
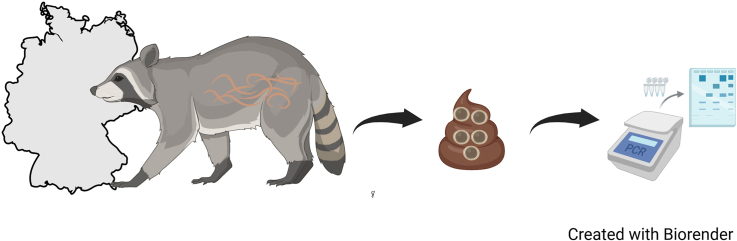

## 1 Introduction

Invasive species can be capable of negatively impact a novel ecosystem.For example, through competition with the endemic fauna for resources or predating on them (Keller et al., 2011; Peter et al., 2024). The raccoon (*Procyon lotor*) is an alien species to Europe, invading new biotopes accompanied with the introduction of uninvited companions, like parasites. One example of such unexpected introductions is the zoonotic nematode *Baylisascaris procyonis*, also called the raccoon roundworm.

Similar to other ascarid parasites, the adults of *B. procyonis* can be found in the small intestine of their definitive hosts: raccoons and, seldomly, dogs or other Procyonidae (Kazacos, 2001). Eggs are then shed in the faeces and can be ingested by other raccoons or small vertebrates. Humans can also get infected through oral ingestion of eggs. *B. procyonis* infection in humans can go from subclinical infection (Weinstein et al., 2017) to severe neurological disease with fatal outcomes, particularly in children (Goldman-Yassen et al., 2022; Sorvillo et al., 2002). Additionally, *B. procyonis* larvae can migrate to the eye (ocular larva migrans) and produce neuroretinitis with vision loss in some cases (Goldberg et al., 1993; Küchle et al., 1993).

Just like its final host, *B. procyonis* originates in North America, from where it was inconspicuously introduced to Europe through Germany during the first half of the last century (Fischer et al., 2016; Frantz et al., 2013), and this country is now a hotspot for the European raccoon population. As the raccoon range expanded, so did its roundworms, although not at an equal rate (Frantz et al., 2024; Heddergott et al., 2020; Osten-Sacken et al., 2018). Currently, *B. procyonis* is present in several countries in this continent, including Austria, France, Germany, Italy, Luxembourg, Norway, the Netherlands, and Poland (Davidson et al., 2013; Duscher et al., 2021; Frantz et al., 2024; Heddergott et al., 2020; Karamon et al., 2014, 2014, 2014; Lombardo et al., 2022; Maas et al., 2022; Rentería-Solís et al., 2018). Presently, *B. procyonis* has been regularly recorded in central, northwest, eastern, and southwestern parts of Germany (Gey, 1998; Heddergott et al., 2020; Osten-Sacken et al., 2018; Peter et al., 2023; Reinhardt et al., 2023; Rentería-Solís et al., 2018). The parasite had been constantly absent from the northeastern regions (Lux and Priemer, 1995; Michler, 2017; Rentería-Solís, 2015; Schwarz et al., 2015) until 2024, when the first reports of *B. procyonis* in the state of Brandenburg were recorded (Kittl et al., 2024; Peter et al., 2024). However, other northern areas remain free from *B. procyonis* (Michler, 2017; Rentería-Solís, 2015). Thus, the objective of this study was to investigate the occurrence of *B. procyonis* in free-ranging raccoons from different areas of Germany.

## 2 Material and methods

### 2.1 Area of study and sample collection

Faecal samples of road-killed and legally hunted raccoons were opportunistically collected between 2019 and 2020 from different federal states of Germany. Sex, weight, and age were recorded whenever possible. Age categorization of juvenile and adult individuals was performed as previously described (Rentería-Solís et al., 2018). Stool samples were stored at −20 °C until further use. Whenever possible, a piece of the small intestine was taken and dissected. Adult ascaroid nematode worms (mostly incomplete) found in the intestinal lumen were collected, and whenever possible, morphologically identified as *B. procyonis* as reported before (Kazacos, 2001; Kazacos and Turek, 1982; Sprent, 1968) and fixed in 4 % formalin.

### 2.2 Scanning Electron Microscopy (SEM) of adult B. procyonis worms

Fixed worms were stained for 60 min with 1 % OsO4 in PBS. Worms were gradually dehydrated in ethanol series (30, 50, 70, 85, 90, 96 % (once), and 100 % (thrice) for 10 min. The parasites were critical-point-dried (Baltec CPD 030, BAL-TEC GmbH, Schalksmühle, Germany) and sputter-coated with 20 nm gold/palladium (Baltec MED 020, BAL-TEC GmbH, Chemnitz, Germany). Worms were visualized using the secondary electron detector of the SEM (Zeiss EVO LS 15 LaB6, Carl Zeiss Microscopy Deutschland GmbH) at an acceleration voltage of 15–25 kV.

### 2.3 Extraction of DNA and molecular detection of B. procyonis DNA using a PCR assay

Mechanical breakdown of *B. procyonis* eggs was done with glass beads using a MagnaLyser (Roche, Germany). Afterwards, extraction of stool DNA was conducted using the QIAamp Fast DNA Stool Mini Kit (Qiagen, Hilden, Germany) according to the manufacturer's instructions. Similarly, genomic DNA (gDNA) from small pieces of tissue from the adult *B. procyonis* found in the intestinal lumen was extracted using the DNeasy Blood and Tissue Kit (Qiagen) following the kit's manual. Afterwards, *B. procyonis* DNA was detected using a published PCR assay protocol (Dangoudoubiyam et al., 2009) aimed to amplify a 146 bp fragment of the *B. procyonis* mitochondrial *cox2* gene. Every reaction consisted of 0.5 μM of each of the primers BpF (forward) 5′-TGAGTTTAGTGATATTCCTGGA-3′and BpR (reverse) 5′-CAGAAGTAATACAAAACCGGAT-3′, 2.5 μL of DreamTaq™ Green Buffer (10x; Thermo Fisher Scientific, Dreieich, Germany), 0.2 μM of each deoxynucleoside triphosphate (dnp), 2 U of DreamTaq Polymerase (Thermo Fisher Scientific), 3 μl of stool DNA or worm gDNA (template) and DNA/nuclease-free water up to a volume of 25 μl. Two additional reactions with DNA/nuclease-free water and a gDNA from an adult nematode previously identified as *B. procyonis* (Rentería-Solís et al., 2018) were added as negative and positive controls, respectively. All PCR reactions described in this study were run in a Biometra Tadvanced® thermal cycler (Analytik Jena, Jena, Germany). PCR products were visualized in an ethidium bromide-stained 1.5 % agarose gel.

### 2.4 Amplification and sequencing of the 18S ribosomal DNA gene (18S gene)

Firstly, a ≈900 bp fragment of the 18S gene specific for the Nematoda phylum was amplified as previously published (Floyd et al., 2005) for all samples. Briefly, each PCR reaction consisted of 2.5 μL of DreamTaq™ Green Buffer (10x; Thermo Fisher Scientific), 0.5 μM of each of the primers Nem18SF (5′-CGCGAATRGCTCATTACAACAGC-3′) and Nem18SR (5′-GGGCGGTATCTGATCGCC-3′) (Floyd et al., 2005), 0.2 μM of each dnp, 2 U of DreamTaq Polymerase (Thermo Fisher Scientific), 3 μL of template (stool DNA collected as described above), and nuclease-free, DNA-free water up to a final volume of 25 μL. Negative and positive controls were added as described above.

PCR conditions consisted of one initial denaturation cycle at 94 °C for 5 min, followed by 35 cycles of denaturation at 94 °C for 30 s, annealing at 54 °C for 30 s, and extension at 72 °C for 1 min; a final extension cycle of 72 °C for 7 min was added. Afterwards, PCR products were purified using the PCR Purification Kit (Jena Bioscience, Jena, Germany) following the kit's instructions. The purified amplicons were bidirectionally Sanger-sequenced (Microsynth Seqlab, Göttingen, Germany) using the primers Nem18SF and Nem18SR.

Generated sequences were analysed using the MEGA X software (version 10.2.6) (Kumar et al., 2018) and blasted in the GenBank database. All the sequences obtained in this study were deposited in the GenBank data base under the accession numbers: PQ846823, PQ846825 to PQ846833, and PQ846835 to PQ846838 (https://www.ncbi.nlm.nhi.gov/genbank/).

### 2.5 Statistical analysis and data visualisation

**Fig. 1:**
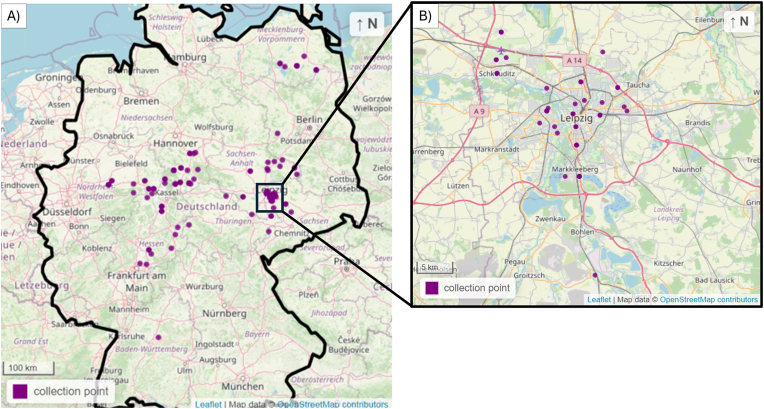
Locations of the collection points for the raccoons investigated in this study. A) Distribution of all the collection areas (in purple) in Germany (delineated in black). B) Amplification of the area within the black square in figure A) which comprises Leipzig metropolitan area and surrounding districts. Map spatial data originated from ©OpenStreetMap with own modifications.

Prevalence results were presented in percentages (%) with their respective confidence intervals (95 % CI), which were calculated with the modified Wald method using the GraphPad Prism software (version 10.1.0, San Diego, USA). Parasite presence was compared to sex, age, and the federal state, using Chi-square or Fisher's exact test (α = 0.05). Mapping of Fig. 1 was done in R (R Core Team, Vienna, Austria) using the leaflet package (Cheng et al., 2024).

## 3 Results

Samples of a total of 166 raccoons were collected in 9 federal states of Germany (Fig. 1). The animals originated from the states of Baden-Wüttemberg (BD) (n = 1), Brandenburg (n = 1), Hesse (n = 22), Lower Saxony (n = 38), Mecklenburg-Western Pomerania (MWP) (n = 13), North Rhine-Westphalia (NRW) (n = 16), Saxony (n = 53), Saxony-Anhalt (SA) (n = 12), and Thuringia (n = 5), with exception of 5 animals for which the collection area was not available. For all the animals, faecal material was available for PCR analysis, and a piece of small intestine was collected from 45 raccoons.

**Fig. 2:**
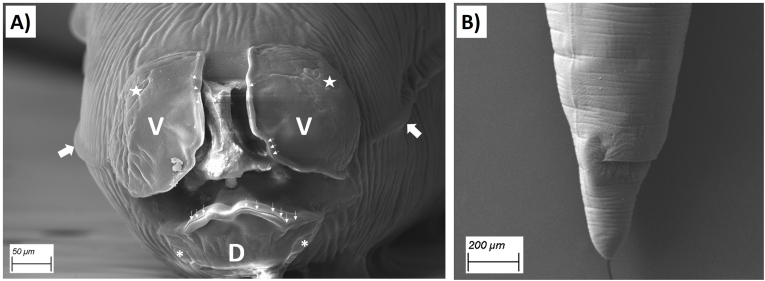
Scanning electron micrographs of a female *B. procyonis* collected in this study. A) Anterior end of the body, apical view of the ventral (V) and dorsal (D) lips, ventral double papilla (stars), dorsal double papilla (asterisk) and labial denticles (small arrows); as well as both lateral alae (thick arrows). B) Posterior end with the vulva opening visible.

**Fig. 3:**
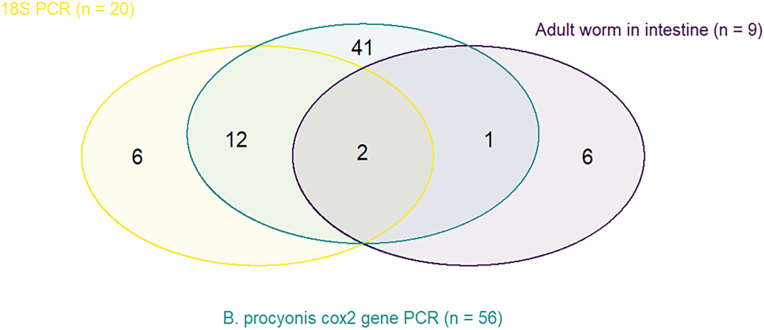
Venn diagram showing the number of positive samples per each PCR assay and presence of adult worms in intestine.

Overall, *B. procyonis* was detected in 68 out of 166 raccoons (41 %, 95 % CI: 33.8–48.6) (Table 1) through either one or more of the diagnostic methods used in the study; namely, presence of adult *B. procyonis* worms, amplification of the *cox2* gene, or amplification and sequencing of the 18S gene (Fig. 3). There were no significant differences in prevalence levels between federal states (χ^2^ = 10.322; df = 8; *p* = 0.24) (Table S1). However, the highest prevalence was found in animals from Saxony-Anhalt with 66.7 % (8 infected raccoons out of 12 samples), but the highest number of positive raccoons, 21 (out of 53 animals) (39.6 %) originated from Saxony. Mecklenburg-Western Pomerania had the lowest number of infected animals with 4 out of 13 (30.8 %). Finally, *B. procyonis* was not found in the few raccoons collected from three federal states, Baden-Württemberg, Brandenburg, and Thuringia (Table S1 of the supplemented material). Adult worms (either complete specimens or pieces thereof) were found in the intestine of 9 raccoons. Three of those were collected and morphologically identified as *B. procyonis* (Fig. 2).

**Table 1:** Prevalence of *B. procyonis* detected in 166 raccoons collected.

Parameter	Group	Positive/total	Percentage (%) of positive animals (95 % CI)	*p*-value
Total of animals investigated		68/166	41.0 (33.8–48.6)	

Sex				
	Female	14/53	26.4 (16.3–39.7)	*p* = 0.01
	Male	54/112	48.2 (37.1–57.4)	

Age				
	Juvenile	9/16	56.2 (33.1–76.9)	p = 0.28
	Adult	59/150	39.3 (31.9–47.3)

Most of the collected raccoons were males (♂: n = 112, 67.4 %; ♀: n = 53, 31.9 %), and in one negative raccoon (0.6 %) sex could not be determined. Males (48.2 %) were infected significantly more (*p* = 0.01) than the females (26.4 %) (Table 1). Regarding age, adults comprised most of the animals collected (90.4 %), while juveniles represented only 9.6 % of the total number of samples. DNA of *B. procyonis* was detected in 59 out of 150 animals (39.9 %) classified as adults and in 9 out of 16 juvenile raccoons (56.2 %). There was no significant difference between the age groups of the animals (*p* = 0.28). A full summary of prevalence rates for each group is shown in Table 1.

Successful partial amplification of the *cox2* gene was assessed in 53 of these animals. Amplicons for a partial region of the 18S gene were produced for 20 of all the samples, from which 14 products were successfully sequenced. The highest identity level for each of these 14 samples (99.86 %–100 %) was to a *B. procyonis* partial sequence (GenBank Acc. No. U94368) collected from a raccoon in the USA (Nadler and Hudspeth, 1998). Additionally, our 14 samples also showed high identity values (99.63 %–99.88 %) to other *Baylisascaris* species, *B. trasfuga* (Acc. Nos. U94369 and JN256988) and *B. schroederi* (Acc. No. JN526988). Finally, a *Baylisascaris* species that also showed high identity levels (99.53 %–99.82 %) with the 14 sequences hereby produced belonged to a *B. ailuri* isolate (Acc. No. JN256991).

## 4 Discussion

In this study, we report the presence of *B. procyonis* in raccoons across Germany with a first report in the northern state of Mecklenburg-Western Pomerania (MWP). Additionally, potential biological risk factors, namely sex and age, were also investigated. To identify the parasite, we used two PCR assays to amplify two different genes: 18S and *cox2*; additionally, we also examined pieces of intestine for the presence of adult worms in 45 animals. Unfortunately, due to the nature of the faecal samples (e.g. low amount of faecal material), flotation tests were not performed for this study. The addition of faecal flotation tests to our study could have increased the sensibility of our results (Page et al., 2025). This was suggested by Page et al. (2025) who compared different methods commonly used to diagnostic *B. procyonis* in raccoons: PCR assays, flotation test, and dissection of complete raccoon intestines. The authors of that study concluded that PCR assays could result in false negatives (Page et al., 2025) and for this reason, the combination of other diagnostic tests would be ideal. We cannot exclude this scenario from our study. Moreover, we only found adult worms in 9 out of 45 intestines collectected. From these 9 animals, only 3 were positive to PCR assay. In our case, the presence of adult worms was limited to only a piece of the intestine since we did not dissect the complete intestinal tract. Therefore, future studies in this population should consider the inclusion of all the previously mentioned diagnostic tests: PCR assays, faecal flotation, and intestine dissection.

More males than females were infected with *B. procyonis* in our study. Such distribution has been a constant outcome in investigations from North America (French et al., 2019; Kazacos, 2001), and it has been similarly reported in other German raccoon populations (Anheyer-Behmenburg, 2013; Reinhardt et al., 2023; Rentería-Solís et al., 2018). A suggested explanation for this sex-biased parasitism is the broader home range of males in comparison to females (French et al., 2019; Page et al., 2009). Page et al. (2009) explained that a larger home range increases the possibilities for males to predate on infected prey. Age is seemingly another skewed factor for raccoons infected with *B. procyonis*. A large number of studies in North America have reported higher prevalence of *B. procyonis* in young animals than in adults (summarized in: French et al., 2019; Kazacos, 2001). Similar results have been described forthe German raccoon population (Anheyer-Behmenburg, 2013; Reinhardt et al., 2023). Only one study on raccoons from Leipzig showed an even distribution between ages (Rentería-Solís et al., 2018), although the population size of that investigation was significantly lower than other studies (Anheyer-Behmenburg, 2013; Reinhardt et al., 2023). Although not significant, in our study, juveniles indeed showed a higher prevalence of the parasite (56.2 %) than adults (39.3 %). Previous studies have suggested that juveniles can be directly infected from the mother and, due to their age, are more susceptible to infection (French et al., 2019; Kazacos, 2001). Adults, on the other hand, ingest infected paratenic hosts (Kazacos, 2001; Page et al., 2008). Additionally, it has been suggested that animals could develop some level of immunity with age after being infected in infancy (French et al., 2019; Kazacos, 2001; Reed et al., 2012). The latter, however, has not been deeply investigated.

In its native North America, the presence of *B. procyonis* varies greatly from region to region. Documented prevalences in the United States of America (USA) go as high as 82 % in the Midwest (Kazacos, 2001), to less than 10 % in the Southeastern state of Florida (Cunningham et al., 2023). The hereby reported presence of 41 % falls within this range. In Germany, *B. procyonis* presence can also differ considerably within the country. Peter et al. (2023) reported the highest prevalence to date in Germany, with 94.9 %. In that project, the authors investigated animals collected mostly from the federal state of Hesse. In fact, the first significantly high prevalence (71 %) was reported by Gey (1998), over 25 years ago, also in raccoons from Hesse. Hesse harbours the densest raccoon population in the country (Fischer et al., 2016; Michler, 2003), which could facilitate parasite transmission amongst them. In our study, we found that 50 % of the animals collected in Hesse were infected with *B. procyonis* (Table S1, supplementary files), which is lower than the studies mentioned above. However, our sample size for that area was considerably smaller (n = 22).

A previous study conducted by Heddergott et al. (2020) reported 43.6 % prevalence, which is very similar to the results we documented in this study. Like in our study, the authors analysed samples from different federal states, including Hesse and MWP. However, they did not find any positive animal in MWP or in Brandenburg. Another study conducted in the northern region of Baden-Württemberg showed a lower presence of the parasite in German raccoons, with 28.7 % of animals infected (Reinhardt et al., 2023). Furthermore, *B. procyonis* prevalence levels in the states of Saxony, Saxony-Anhalt, and Thuringia ranged from 39 % (Peter et al., 2024) to 88.9 % (Winter, 2005). Our sampled population also comprised animals from these states. Almost 40 % of the animals that we collected in Saxony were positive for *B. procyonis* as well as 66.7 % from Saxony-Anhalt (Table S1, Supplementary data). Interestingly, a previous report from Leipzig, Saxony, showed a prevalence of 75 % (Rentería-Solís et al., 2018). However, the sample size of that study was significantly smaller than ours.

Historically, two distinct foci of high dense populated regions can still be found in the German raccoon populations: one in the centre of the country, mostly in the state of Hesse, and one in the northeast, covering the states of Berlin, Brandenburg, and MWP (Fischer et al., 2016; Hohmann and Bartussek, 2005). *B. procyonis* had not been reported in the northeastern raccoons (Heddergott et al., 2020; Lux and Priemer, 1995; Michler, 2017; Rentería-Solís, 2015) until very recently, when two different studies detected *B. procyonis* in animals from Brandenburg (Kittl et al., 2024; Peter et al., 2024). Similarly, our study shows presence of *B. procyonis* in MWP for the first time, with 4 infected animals (out of 13). These positive raccoons were collected in the region of Müritz, either close to or within the Müritz National Park. The raccoon population of this region has been comprehensively studied for almost 20 years (Michler, 2003, 2017; Michler et al., 2023). During all this time, *B. procyonis* was not found in these raccoons, and the population was believed to be free of this parasite (Michler, 2017; Rentería-Solís, 2015), until now.

Since the last decade, notable efforts have been made to elucidate *B. procyonis* and the raccoon's journey of introduction, establishment, spread, and colonization history in Europe with Germany as a pivotal research area (Fischer et al., 2016; Frantz et al., 2013; Heddergott et al., 2020, 2023; Maas et al., 2022; Osten-Sacken et al., 2018). Using microsatellite analysis, Osten-Sacken et al. (2018) produced for the first time a geographic distribution of genetic clusters for the raccoon and *B. procyonis* populations in Germany. While the raccoon population in Germany was found to be genetically diverse, *B. procyonis* lack of haplotype variety was noticeable. Their data was later enriched by the study from Heddergott et at. (2020); however, the parasite genetic diversity remained the same. Thanks to these investigations, it is known that the raccoons from MWP are part of the “Brandenburg” genetic cluster, along with animals from Brandenburg and northeastern Saxony-Anhalt. These studies also reported the absence of *B. procyonis* in the raccoons from MWP. Unfortunately, we did not investigate the genetic diversity of the *B. procyonis* found in MWP. Similarly, the haplotype of the *B. procyonis* specimens reported in the neighboring state of Brandenburg the previous year (2024) was also not studied (Kittl et al., 2024; Peter et al., 2024). Whether the parasite is moving north, or we are facing a new separate introduction, would need to be investigated. Microsatellite analysis of population genetics is a suitable option for our and other samples, and should be explored further to enhance the current *B. procyonis* population genomics research (Heddergott et al., 2020, 2023; Maas et al., 2022; Osten-Sacken et al., 2018)

*B. procyonis* research in Germany and other areas of Europe is still in its infancy. However, the past and current efforts to investigate and understand this parasite have provided important insights into its biology, epidemiology, and migration history on this continent (Anheyer-Behmenburg, 2013; Frantz et al., 2024; Gey, 1998; Heddergott et al., 2020, 2023; Osten-Sacken et al., 2018; Peter et al., 2024; Reinhardt et al., 2023; Rentería-Solís et al., 2018). Continuous monitoring of parasite and host distribution, enrichment of the genetic population database, risk assessment investigations, epidemiology of *B. procyonis* in non-raccoon hosts and the environment should provide us with tools to not only follow the parasite's movement to the north, but also to control it.

## CRediT authorship contribution statement

**Zaida Rentería-Solís:** Writing – review & editing, Writing – original draft, Visualization, Validation, Supervision, Software, Resources, Project administration, Methodology, Investigation, Funding acquisition, Formal analysis, Data curation, Conceptualization. **Luis Flores:** Writing – review & editing, Investigation, Data curation. **Torsten Langner:** Writing – review & editing, Resources, Investigation, Data curation. **Sandra Gawlowska:** Investigation. **Thomas Grochow:** Writing – review & editing, Visualization, Resources, Investigation. **Simone Fietz:** Visualization, Resources, Funding acquisition. **Stefan Birka:** Supervision, Resources, Funding acquisition, Conceptualization. **Nina Król:** Writing – review & editing, Funding acquisition, Formal analysis, Conceptualization. **Anna Obiegala:** Writing – review & editing, Supervision, Resources, Investigation, Funding acquisition, Data curation, Conceptualization.

## Ethical Board Statement

Animals used in this study were either found dead or legally hunted in accordance with German legislation. Therefore, an Ethical Board Statement is not required for conducting this study.

## Funding

This work was partially funded by a grant from the Leipzig veterinary junior scientist support program financed by the *Freundeskreis Tiermedizin* and the 10.13039/501100022271Faculty of Veterinary Medicine (grant to A.O.), the funding institutions had no part in this study. Article processing charges were covered thanks to the DEAL Project.

## Declaration of competing interests

The authors declare no conflict of interests.
