# Supplementary material for: The conquest of the north continues: Baylisascaris procyonis in free-ranging invasive raccoons (Procyon lotor) from Germany, including a first report in the northeastern state of Mecklenburg-Western Pomerania
Source: Int J Parasitol Parasites Wildl. 2025 Sep 17;28:101139. doi: 10.1016/j.ijppaw.2025.101139 (PMC12481708; doi:10.1016/j.ijppaw.2025.101139)
Supplement: Multimedia component 1 [file mmc1.docx]

| **Federal state** | **Number of positive animals/number of animals in total** | **Percentage (%) (95% CI)** | ***p* value** |
| --- | --- | --- | --- |
| Baden-Württemberg | 0/1 | 0 | *p* = 0.24 |
| Brandenburg | 0/1 | 0 |  |
| Hesse | 11/22 | 50 (30.7 – 69.3) |  |
| Mecklenburg-Western Pomerania | 4/13 | 30.8 (12.35 – 58.0) |  |
| Lower-Saxony | 17/38 | 44.7 (30.1 – 60.3) |  |
| North Rhine-Westphalia | 5/16 | 31.2 (13.9 – 55.8) |  |
| Saxony | 21/53 | 39.6 (27.8 – 53.1) |  |
| Saxony-Anhalt | 8/12 | 66.7 (38.8 – 86.4) |  |
| Thuringia | 0/5 | 0 |  |
